# Energy balance following diets of varying fat content: metabolic dysregulation in a rodent model of spinal cord contusion

**DOI:** 10.14814/phy2.14207

**Published:** 2019-08-27

**Authors:** Kwamie K. Harris, Alexandra R. Himel, Brittany C. Duncan, Raymond J. Grill, Bernadette E. Grayson

**Affiliations:** ^1^ Department of Neurobiology and Anatomical Sciences University of Mississippi Medical Center Jackson Mississippi

**Keywords:** Metabolic syndrome, obesity, spinal cord injury

## Abstract

Within the spinal cord injured (SCI) population, metabolic dysfunction may be exacerbated. Models of cord injury coupled with metabolic stressors have translational relevance to understand disease progression in this population. In the present study, we used a rat model of thoracic SCI at level T10 (tSCI) and administered diets comprised of either 9% or 40% butterfat to create a unique model system to understand the physiology of weight regulation following cord injury. SCI rats that recovered on chow for 28 days had reduced body mass, lean mass, and reduced fat mass but no differences in percentage of lean or fat mass composition. Following 12 weeks on either low‐fat diet (LFD) or high‐fat diet (HFD), SCI rats maintained on LFD did not gain weight at the same rate as SCI animals maintained on HFD. LFD‐SCI had reduced feed conversion efficiency in comparison to Sham‐LFD whereas tSCI‐HFD were equivalent to Sham‐HFD rats. Although SCI rats still maintained lower lean body mass, by the end of the study HFD‐fed rats had higher body fat percentage than LFD‐fed rats. Macronutrient selection testing demonstrated SCI rats had a significant preference for protein over Sham rats. Analysis of metabolic cage activity showed tSCI rats had elevated energy expenditure, despite reduced locomotor activity. Muscle triglycerides and cholesterol were reduced only in LFD‐tSCI rats. These data suggest that consumption of HFD by tSCI rats alters the trajectory of metabolic dysfunction in the context of spinal cord disease progression.

## Introduction

Innovations to specialized healthcare have greatly improved the longevity of persons with injury to the spinal cord (Strauss et al. 2006). As the average age of this injured subpopulation continues to rise, the potential for the various comorbidities of Metabolic Syndrome (MetS) also increases (Maruyama et al. 2008). In fact, persons with SCI are at increased risk for dyslipidemia, cardiovascular disease, and glycemic dysregulation, all contributing factors to MetS (Akkurt et al. 2017; Alves et al. 2017; Berg‐Emons et al. 2008). Development of MetS can complicate long‐term care for these patients. Therefore, understanding how and why MetS develops in this vulnerable population is important for improving quality of life for these individuals.

Obesity is a disorder of energy balance where intake and output are mismatched resulting in increased storage of energy as adipose tissue. The rate of obesity among subpopulations of SCI individuals varies from 25 to 57% (Gater 2007; Gorgey et al. 2014; Gater et al. 2019) which is somewhat higher than the national range 22–35% (Ogden et al. 2013). Reduced ability to exercise and increased consumption of calories may contribute to this increased risk. Altered neural connectivity, hormones, and inflammatory factors may also add to this disparity, but no specific factors have been identified to‐date that support the elevated risk for MetS.

SCI individuals suffer from increased incidence of two subcategories of obesity 1) “visceral obesity” or obesity primarily centered around the abdominal region and 2) “sarcopenic obesity” or obesity due to reduced lean mass or quality (Stenholm et al. 2008). Visceral obesity has great relevance to the obesity epidemic because of its high association with insulin resistance, type 2 diabetes and cardiovascular disease (Bays et al. 2013). Sarcopenic obesity is the result of significant muscle atrophy due to reduced ability to ambulate and exercise (Stenholm et al. 2008). One study estimates that following SCI anywhere from 27 to 65% of the muscle fibers atrophy in the first 6–18 months (Castro et al. 2000). This loss of muscle mass also contributes to the reduced basal metabolic rate and energy expenditure (Castro et al. 2000). Methods to estimate energy expenditure following spinal injury suggest that resting metabolic rate is 14–27% lower in persons with SCI compared to those without injury (Buchholz and Pencharz 2004) and also worse in injuries with complete transections located higher in the cord (Mollinger et al. 1985).

Along with the obvious reduced energy output in persons with SCI, caloric intake in persons with SCI may be different than noninjured individuals. Recent work concerning food intake patterns of persons with SCI report increased total food intake in comparison to individuals who are immobile for other reasons (Pellicane et al. 2013; Farkas et al. 2019). Food intake pattern analysis ofoverweight or obese persons with SCI suggests that overall calories are distributed within the acceptable range for energy needs but that there is increased consumption of calories from fat (Silveira et al. 2019).

In the present work, we used a rodent model of thoracic spinal contusion at level T10 and then administered two diets (9% fat vs. 40% fat) to create a unique model system to understand the physiology of body‐weight regulation following cord injury. The two diets were equivalent in protein content but varied in fat and carbohydrate content. We hypothesized that tSCI rats would overconsume the high‐fat diet (HFD) and that this would exacerbate the metabolic dysfunction (i.e., obesity) of the SCI animals. We report measures of body weight gain, body composition, food intake, macronutrient preference and metabolic rate to understand how these diets with two different concentrations of fat affect long‐term injury recovery.

## Methods

### Animals

All procedures for animal use complied with the *Guidelines for the Care and Use of Laboratory Animals* by the National Research Council and were reviewed and approved by the University of Mississippi Medical Center Institutional Animal Care and Use Committee (IACUC #1469) and the US Army’s Animal Care and Use Review Office (ACURO). In conducting research using animals, the investigators adhered to the laws of the United States and regulations of the Department of Agriculture.

Male, Long Evans rats (250–300 g, approximately 12 weeks old) (Envigo, Indianapolis, IN) (*N* = 39) were initially multiply housed and maintained in a room on a 12/12‐h light/dark cycle at 25°C and 50–60% humidity with ad libitum access to water and standard chow (#8640, Envigo, 3.0 kcal/g; 17% fat, 54% carbohydrate, 29% protein). Rats were acclimated to the vivarium for 1 week prior to injury. Rats were assigned to either Sham‐laminectomy (Sham) (*N* = 17) or thoracic spinal cord injury (tSCI) (*N* = 22) groups in a counterbalanced fashion based on body weight on the day prior to the start of surgery. Surgery was performed and animals were allowed to recover for 28 days following surgery while consuming standard chow. Rats were singly housed from the time of surgery to the end of the study. After 28 days, rats were switched to one of two protein‐matched diets: high‐fat diet (HFD) (#D03082706, Research Diets, New Brunswick, NJ, 4.54 kcal/g; 40% fat, 45% carbohydrate, 15% protein) or low‐fat diet (LFD) (#D03082705, Research Diets, New Brunswick, NJ, 3.81 kcal/g, 9% fat, 76% carbohydrate, and 15% protein) for 12 weeks until the remainder of the study totaling 16 weeks. The number of animals in each group at the end of the study were: Sham‐LFD (*N* = 8), tSCI‐LFD (*N* = 10), Sham‐HFD (*N* = 8) and tSCI‐HFD (*N* = 8). One Sham died of unknown causes after 2 months. Euthanasia was performed due to some animals’ autophagic behavior from significant neuropathic pain that is common in Long Evans rats following SCI (Mills et al. 2001; He and Nan 2016).

### Surgical procedures

All surgical procedures were performed on animals that were deeply anesthetized using 5% isoflurane with a gradual decrease to 2.5%. tSCI surgeries were performed as previously described (Scheff et al. 2003). During the surgery, the animal was placed on a heating pad set to 41°C. The heating pad was removed during the impact portion of the surgery and replaced for the suture portion of the surgery. Incisions were made on the animals’ dorsal skin and overlying muscles and the vertebral column was exposed. A laminectomy was performed at thoracic level 10 (T10) and the vertebral column was stabilized using Anderson Forceps that grasp the ventral surface of the lateral spinous processes at vertebral levels T9 and T11. Using an Infinite Horizon Spinal Impactor Device (Precision Systems and Instrumentation, LLC, Fairfax Station, VA), moderate contusion injury was delivered to the T10 spinal cord using 150 kdynes of force with a 1 sec dwell. The area was inspected for bruising and the digital trace was observed to ensure there was no bone obstruction and therefore an appropriate injury. The dura mater remained closed for the entire duration of SCI surgeries. Immediately following tSCI, the overlying muscles were sutured and the skin was securely closed using stainless steel wound clips.

Sham‐laminectomy surgery was performed consisting of incisions to the animals’ dorsal skin exposing the musculature and vertebral column. A laminectomy was performed at T10 vertebrae and then the overlying muscles were sutured and the skin securely closed using stainless steel wound clips.

### Postoperative care

Animals received one dose of buprenorphine SR (Sustained Release) (1.0–1.2 mg/kg SQ (ZooPharm, Laramie, WY) and 72 h later, single‐dose buprenorphine for postsurgical pain management (0.025 mg/kg, twice daily for a period of 2 days, then as needed). Animals also receive (1) antibiotic, naxcel (5 mg/kg SQ, Zoetis, NJ) once daily for a period of 5 days, (2) 3–5 mL of 0.9% saline, twice daily for a period of 3 days to ensure hydration. Beginning the day of spinal injury, each rat’s urinary bladder was manually expressed two to three times daily until the animal recovered the ability to void its bladder. The T10 contusion disrupts the supraspinal pathways that are responsible for bladder voiding. In our hands, control of neurogenic bladder function returns in approximately 14 days. As a rule, bladder care was discontinued for an animal when it exhibited an already‐voided bladder on two consecutive bladder care sessions.

### Hindlimb locomotor function assessment

Hindlimb locomotor function was assessed using the Basso, Beattie, and Bresnahan (BBB) open‐field locomotor scale (Basso et al. 1995). BBB scores were initially assessed on days 1, 7, 14 and 28 postinjury. Only animals who achieved a 1 or lower on day 1 postinjury were allowed to continue in the study. Following diet inductions on day 28, BBB was tested at weeks 8, 12, and 16 on diet. Briefly, the rat was placed into the open field and allowed to move freely for approximately 4 min. Movement and articulation of the joints of each hindlimb were scored using a scoring sheet. A 21‐point BBB Open‐Field Rating Scale was used for the determination of intact locomotor behavior. When an animal reached the score of 21, it was no longer tested. For each animal, the locomotor scores for both hindlimbs were averaged to produce one score per test session.

### Body weight and composition

Following surgery, animals were weighed daily for the first 14 days and then weekly thereafter. Lean and fat mass were analyzed using Echo Magnetic Resonance Imaging (echoMRI) (EchoMedical Systems, Houston, TX) at weeks 4 (prior to start of diet), 8, 12, and 16.

### Blood collection and measurements

During postinjury week 12, tail vein blood collection was performed. This time point was chosen prior to the complex TSE scheduling. Tail bleeds occurred within 2 h of lights‐on to obtain plasma for ad libitum‐fed analyses. Food was then removed from the animals for 24 h and the following morning within 2 h of lights‐on, an additional fasting plasma sample was procured. Non‐esterified fatty acids (NEFA), phospholipids, *β*‐hydroxybutyrate, total plasma triglycerides, and total plasma cholesterol were measured. During postoperative week 15, animals were fasted for ~6 h following lights‐on. Baseline blood glucose was measured using an AccuChek glucometer. The remaining analytes were measured from terminal trunk blood that was obtained after food was removed and 6–8 h after lights‐on (Table 1).

**Table 1:** Plasma analytes.

Plasma analyte	Time	Sham‐LFD	tSCI‐LFD	Sham‐HFD	tSCI‐HFD	Statistics
		Mean ± SEM	Mean ± SEM	Mean ± SEM	Mean ± SEM	
NEFA (mEq/L) (fed)	wk12	0.79 ± 0.03	0.85 ± 0.10	0.89 ± 0.08	1.07 ± 0.08	*P* (diet) = 0.07
NEFA (mEq/L) (fasted)	wk12	0.94 ± 0.06	1.05 ± 0.05	1.08 ± 0.09	0.96 ± 0.06	
Phospholipids (mg/dL) (fed)	wk12	223.50 ± 9.44	208.00 ± 23.72	198.20 ± 18.22	238.90 ± 25.28	
Phospholipids (mg/dL) (fasted)	wk12	95.53 ± 6.30	105.70 ± 9.28	94.18 ± 3.00	80.85 ± 4.10	*P* (diet) = 0.069
Β‐hydroxybutyrate (mg/dL) (fed)	wk12	0.93 ± 0.14	1.36 ± 0.20	1.33 ± 0.22	1.22 ± 0.20	
B‐hydroxybutyrate (mg/dL) (fasted)	wk12	9.44 ± 1.33	10.47 ± 0.90	11.71 ± 0.89	13.41 ± 1.23	*P* (diet) < 0.05
Total cholesterol (mg/dL) (fed)	wk12	131.20 ± 6.81	115.60 ± 9.83	111.30 ± 6.15	109.10 ± 4.78	*P* (diet) = 0.0842
Total cholesterol (mg/dL) (fasted)	wk12	103.30 ± 8.21	87.97 ± 6.93	76.65 ± 4.78	72.19 ± 4.24	*P* (diet) < 0.01
Total triglycerides (mg/dL) (fed)	wk12	429.30 ± 50.29	415.50 ± 67.50	733.40 ± 120.30	689.50 ± 107.60	*P* (diet) < 0.01
Total triglycerides (mg/dL) (fasted)	wk12	169.90 ± 24.48	152.20 ± 35.16	184.70 ± 23.70	149.00 ± 22.62	
Plasma glucose (fasted)	wk15	128.10 ± 9.10	130.30 ± 3.81	130.60 ± 5.83	127.40 ± 4.92	
C‐Peptide (pM) (fasted)	wk16	580.30 ± 145.70	507.40 ± 104.40	928.30 ± 130.20	720.90 ± 109.40	*P* (diet) < 0.05
Insulin (ng/mL) (fasted)	wk16	3.18 ± 0.52	3.57 ± 0.79	8.01 ± 1.11	4.90 ± 1.82	*P* (diet) < 0.05
Leptin (ng/mL) (fasted)	wk16	10.94 ± 2.15	8.57 ± 2.45	16.88 ± 1.99	12.25 ± 1.84	*P* (diet) < 0.05
Hepatic triglycerides (mg/dL)	wk16	529.90 ± 70.12	555.40 ± 70.18	751.50 ± 64.67	645.80 ± 66.81	*P* (diet) < 0.05
Hepatic cholesterol (mg/dL)	wk16	138.50 ± 3.41	148.30 ± 4.45	153.90 ± 4.53	151.20 ± 5.27	*P* (diet) = 0.0519
Muscle triglycerides (mg/dL)	wk16	512.6 ± 99.17	238.5 ± 46.59	478.6 ± 89.82	499.1 ± 126	Sham‐LFD versus tSCI‐LFD, *P* > 0.05
Muscle cholesterol (mg/dL)	wk16	200.5 ± 20.91	145.8 ± 6.601	195.7 ± 22.24	193.6 ± 22.87	Sham‐LFD versus tSCI‐LFD, *P* > 0.05
Plasma ALT (fasted)	wk16	95.93 ± 7.755	88.69 ± 7.011	88.69 ± 7.011	88.81 ± 7.977	
Plasma AST (fasted)	wk16	24.51 ± 2.213	25.35 ± 1.437	27.47 ± 1.24	29.89 ± 2.575	*P* (diet) < 0.05

### Feed conversion efficiency (FCE) calculation

FCE was calculated using the change in body weight over the first week divided by the kcal consumed during the first week.

### Macronutrient selection testing

During week 12 postinjury, three pure macronutrient diets, (Harlan Teklad; TD.02521[carbohydrate], TD.02522[fat], and TD02523[protein]) were presented in separate containers simultaneously for 4 days. Animals were acclimatized during the first 48 h to the new diets. The total amount consumed of each macronutrient during the final 24 h period was converted to kcal consumed and then a percentage for each macronutrient was reported.

### Metabolic system monitoring

During postinjury weeks 13–15, rats were placed in special metabolic cages (AccuScan Instruments Inc, Columbus, OH) in a staggered fashion for about 64 h. The first 24 h were considered an acclimatization period and only the data collected during the second 24 h was used in the calculations. Rats were housed individually in an acrylic cage (16 × 24 × 17 cm) equipped with oxygen sensor to measure oxygen consumption (VO_2_) and infrared beams to determine motor activity. VO_2_ was measured for 2 min at 10‐min intervals using a Zirconia oxygen sensor. This system also measured carbon dioxide production (VCO2). Respiratory quotient was calculated as VCO_2_/VO_2_ (Evans et al. 2004). Heat production was derived from the following formula (4.33 + (0.67 * RQ) * VO_2_ * weight (grams) * 60. Energy expenditure was mathematically calculated post hoc according to Weir using the following equation: total EE (kJ h^−1^)=16.3 × *V*O_2_ (L h^−1^) + 4.57 × VCO_2_ (L h^−1^) (Weir 1949). Animal motor activity was determined using infrared light beams mounted in the cages in *X*, *Y*, and *Z* axes. Precise measurements of food and liquid consumption were taken manually every 24 h.

### Tissue harvest

During week 16 postinjury, rats were euthanized by conscious decapitation starting at 6 h following the onset of the light cycle. Tissues excised include terminal plasma, liver, and gastrocnemius. Tissue was flash frozen with methylbutane on dry ice and then stored in −80°C until further processing.

### Plasma analytes

All blood was collected in heparin or EDTA coated tubes, processed for plasma and stored at −80°C until further use. Plasma was diluted 1:20 in saline in order to measure triglycerides (#TR22421, Infinity Triglyceride Reagent, Thermo Scientific, Waltham, MA) and cholesterol (Infinity Cholesterol, #TR13421, Thermo Scientific, Waltham, MA). Additional analytes measured using enzymatic assays include AST (A7561‐150, Pointe Scientific) and ALT (A7526‐150), *β*‐hydroxybutyrate (#SBHR‐100, Fisher Scientific, Waltham, MA), and NEFA (Wako Diagnostics, Richmond, VA). All assays were performed according to the manufacturers’ specifications. Nonesterified fatty acids (Wako Diagnostics, Richmond, VA), hydroxybutyrate, phospholipids, and C‐peptide were all measured using the University of Cincinnati MMPC core.

### Hepatic and gastrocnemius triglyceride and cholesterol determination

Hepatic triglycerides and cholesterol were measured using Pointe Scientific Triglyceride reagent set (#T7532‐120) and calibrator (#T7532‐STD) and Pointe Scientific Cholesterol reagent set (#C75‐10‐120) and calibrator (#C7509‐STD) using 50 mg of fresh frozen liver or gastrocnemius muscle tissue according to manufacturer’s specifications.

### Statistical analyses

All statistical analyses were performed using GraphPad Prism version 7.02 (GraphPad Software, San Diego, CA). Differences between two groups were assessed by using unpaired Student's *t* test and two‐tailed distribution. Statistical significance was determined with two‐way analysis of variance followed by Tukey’s post hoc test for variables of injury and diet. To observe time‐wise differences, repeated measures, two‐way ANOVA (variables: Sham‐LFD, sham‐HFD, tSCI‐LFD, and tSCI‐HFD) with Tukey post hoc test was used. All results are given as means ± SEM. Results were considered statistically significant when *P* < 0.05.

## Results

### Changes in body mass and composition during first 28 days

**Figure 1:**
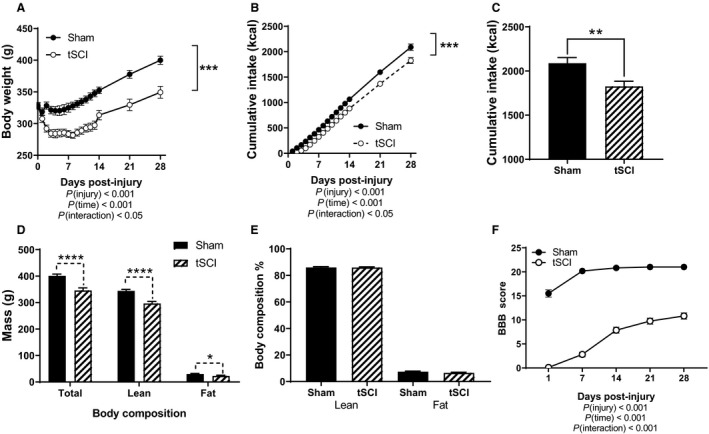
Parameters during initial four weeks post‐injury. (A) Daily body weight during first 14 days and then weekly measured in grams (g). (B) Cumulative chow intake (kcals). (C) Total cumulative chow intake during first 28 days. (D) Body composition analysis in grams (g) measured by EchoMRI: total, lean and fat. (E) Lean and fat body composition by percentage of body weight. (F) BBB locomotor score at 1, 7, 14, 21 & 28 days post‐injury. Data is presented as mean ± SEM. Statistical significance was determined with two‐way analysis of variance followed by Tukey’s *post hoc* test (A,B,F) and Student’s *T* test (C–E). *P < 0.05, **P < 0.01, ***P < 0.001, ****P < 0.0001. *N* = 17 and 22/group.

Male tSCI rats lost a significant amount of body weight during the first 7 days postinjury in comparison to Sham rats (main effect of injury, *P* < 0.001, main effect of time, *P* < 0.001, interaction time × injury, *P* < 0.05) (Fig. 1A). Rats having received tSCI returned to their preinjury weight by approximately 21 days postinjury (Fig. 1A). Although the tSCI rats increased in body weight at a parallel trajectory to sham‐injured animals, they did not exhibit catch‐up body weight gain during their early recovery period (Fig. 1A). tSCI rats consumed fewer calories from chow during the first 28 days after injury in comparison to Sham rats (main effect of injury, *P* < 0.001 and main effect of time, *P* < 0.001¸ interaction time × injury, *P* < 0.05) (Fig. 1B and C). Overall the tSCI rats weighed less than the Sham rats (*P* < 0.0001) (Fig. 1D), had less lean body mass (*P* < 0.0001) (Fig. 1D), and less fat mass (*P* < 0.05) (Fig. 1D). However, the percentage of lean and fat mass normalized to body weight between the two groups did not vary among the groups (Fig. 1E). Thus, as the Sham animals increased proportionately in size during this time frame, tSCI animals grew proportionately but remained reduced in overall size.

BBB testing to assess locomotor function was first performed on day 1 postinjury. For tSCI rats, the average BBB score was approximately 0 and rose to a score of 11 for both hindlimbs on day 28 postinjury (Fig. 1F). On day 1, Sham rats had a score of approximately 15 which was restored to 21 on day 7 post injury (main effect of injury, *P* < 0.001, main effect of time, *P* < 0.001) (Fig. 1F).

### Changes in body mass and composition during 12‐week diet phase

**Figure 2:**
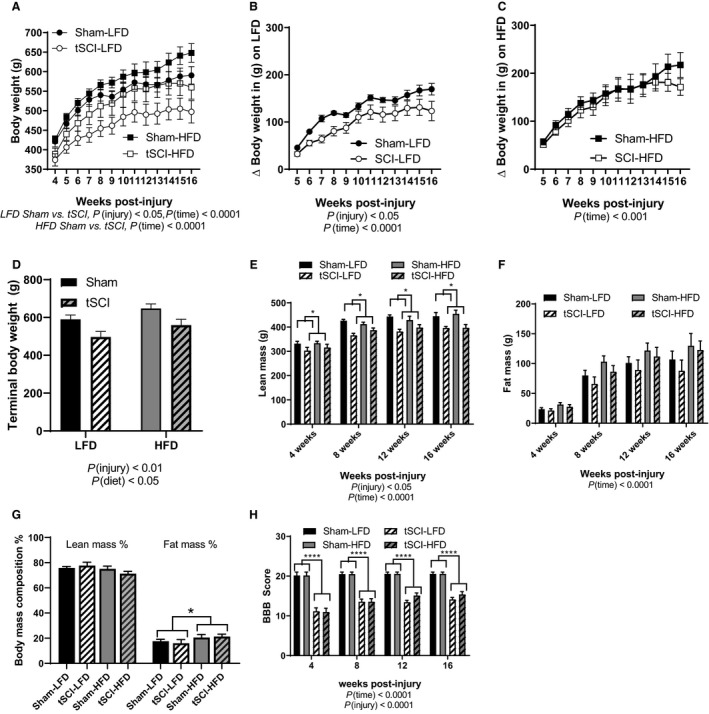
Body weight and composition. (A) Weekly body weight in grams for Sham and tSCI animals placed on LFD and HFD. (B) Body weight change of Sham and tSCI animals placed on LFD. (C) Body weight change of Sham and tSCI animals placed on HFD. (D) Terminal body weight of rats in the study. (E) Lean body mass in grams measured by EchoMRI over the 12 weeks on diet. (F) Fat body mass in grams measured by EchoMRI over the12 weeks on diet. (G) Terminal lean and fat body composition %. (H) BBB locomotor score though 16 weeks post‐injury. Data is presented as Mean ± SEM. Statistical significance was determined by repeated measures, two‐way analysis of variance followed by Tukey’s post hoc test. *P < 0.05, ****P < 0.0001. *N *= 7–11/group.

Following the 28‐day recovery period, rats were placed either on LFD or HFD (Fig. 2A). Irrespective of group, all the rats continued to gain weight throughout the 12‐week time frame. LFD‐fed tSCI animals continued to have a significantly reduced body weight in comparison to Sham (main effect of injury, *P* < 0.05; main effect of time, *P* < 0.0001) (Fig. 2A). HFD‐fed tSCI increased in body weight in a trajector similar to Sham (main effect of time, *P* < 0.0001) (Fig. 2A). tSCI animals on LFD displayed an attenuated body weight change from the time of the diet induction to the end of the 12 weeks of diet induction (main effect of injury, *P* < 0.05, main effect of time, *P* < 0.0001) (Fig. 2B). However, tSCI animals placed on HFD had similar body weight change to the Sham‐HFD animals (main effect of time, *P* < 0.001) (Fig. 2C). This suggests that the altered macronutrient content of the HFD caused accelerated weight gain for the tSCI rats in comparison to the LFD‐tSCI rats but similar to Sham‐HFD. At the end of the study, tSCI rats remained lighter than Sham, and LFD‐fed animals continued to weigh less overall then HFD (main effect of injury, *P* < 0.01, main effect of diet, *P* < 0.05) (Fig. 2D). Overall, tSCI rats continued to have less lean body mass than Sham rats (main effect of injury, *P* < 0.01) (Fig. 2E) following 12 weeks of a LFD/HFD feeding. Although overall body fat increased over time for all animals, there was no difference in body fat as a result of diet or injury (Fig. 2F) only time *P* < 0.0001. When this was normalized for body weight, there was no difference in the lean mass percentage by diet or injury (Fig. 2G). However, the HFD‐fed Sham and tSCI had a higher percentage of fat in comparison to LFD‐fed Sham and tSCI, (main effect of diet, *P* < 0.05) (Fig. 2G). We also assessed BBB scores every 4 weeks. Whether on LFD or HFD, tSCI animals had relatively similar BBB scores that continued to increase to a final mean score of 14.5 during week 16 of the (Fig. 2H). On the other hand, sham‐injured rats received an average BBB rating of 21 by day 7 postinjury and remained at this score for the subsequent 16 weeks of the study (main effect of injury, *P* < 0.0001, main effect of time, *P* < 0.0001) (Fig. 2H).

### Diet consumption

**Figure 3:**
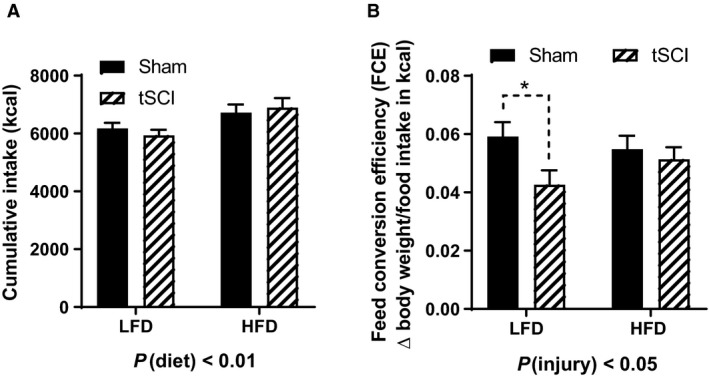
Calorie consumption. (A) Cumulative food intake reported in kilocalories. (B) Feed conversion efficiency measured as weight gain over weekly kcal intake first week on LFD or HFD. Data is presented as mean ± SEM. Statistical significance was determined by two‐way analysis of variance followed by Tukey’s post hoc test and Student’s T test (B). *P < 0.05. *N* = 7–11/group.

Overall, the impact of injury on food intake was diminished such that there was only a significant effect of diet on the number of calories ingested, that is, HFD‐fed animals consumed more calories than LFD‐fed rats (main effect of diet, *P* < 0.01) (Fig. 3A). We performed a feed conversion efficiency (FCE) calculation to more closely analyze the first week on the respective new diets and there was a significant main effect of injury, (*P* < 0.05). We determined that the tSCI‐LFD rats were not converting the consumed calories into body mass gain compared to Sham‐LFD rats (*P* < 0.05), and that the HFD‐fed tSCI rats were converting the calories into body weight at an equal rate to the Sham‐HFD rats (Fig. 3B).

### Metabolic cage analysis

**Figure 4:**
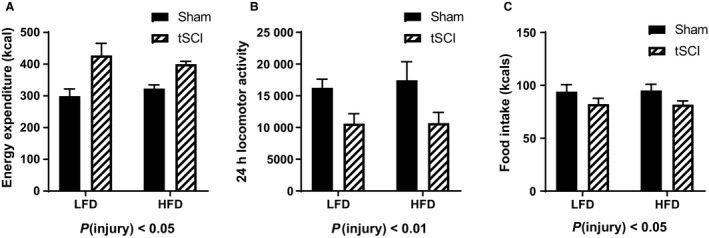
Metabolic cage analysis. (A) Calculated energy expenditure in kcal. (B) 24 h locomotor activity. (C) Food intake during metabolic monitoring in kcal. Data is presented as mean ± SEM. Statistical significance was determined by two‐way analysis of variance followed by Tukey’s post hoc test. *N* = 7–11/group.

Placing the animals in metabolic cages, we determined that tSCI rats had significantly increased energy expenditure (main effect of injury, *P* < 0.05) (Fig. 4A). Twenty‐four hours locomotor activity was lower in tSCI animals as result of injury (main effect of injury, *P* < 0.01) 1 (Fig. 4B). During this time, food intake was reduced in the tSCI rats in comparison to Sham (main effect of injury, *P* < 0.05) (Fig. 4C).

### Macronutrient selection test

**Figure 5:**
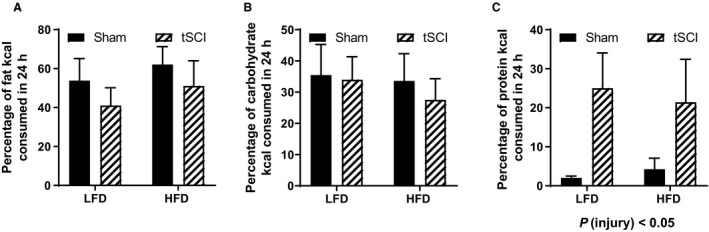
Macronutrient Selection Test. (A) Macronutrient selection test percent of fat kcal consumed. (B) Macronutrient selection test percent of carbohydrate kcal consumed. (C) Macronutrient selection test percent of protein kcal consumed. Data is presented as mean ± SEM. Statistical significance was determined by two‐way analysis of variance followed by Tukey’s post hoc test. *N* = 7–11/group.

During postinjury week 12, we performed a macronutrient selection test, offering pure fat, carbohydrates, and protein. When normalized to each animal's total kcal content, there was no difference in the percent of fat (Fig. 5A) or carbohydrate consumed (Fig. 5B). tSCI rats consumed significantly more percentage of protein then Sham rats during the test period (main effect of injury, *P* < 0.05) (Fig. 5C).

### Plasma analytes

We wanted to compare blood obtained during the ad libitum fed and 24 h fasted state in order to determine the differences in energy utilization. We observed no differences in fed or fasted NEFA and phospholipids levels among groups (Table 1) and elevated fasting levels of *β*‐hydroxybutyrate, *P* (diet) < 0.05 in HFD‐fed animals (Table 1). Total triglycerides were significantly increased in HFD‐fed animals in comparison to LFD‐fed animals in the fasting condition, *P* (diet) < 0.01 (Table 1) whereas total cholesterol levels were reduced in HFD‐fed animals after 24 h of fasting, *P* (diet) < 0.01 (Table 1). No differences among groups were identified in plasma glucose measured 15 weeks postinjury (Table 1). Fasting plasma insulin, C‐peptide, a marker of insulin production, and leptin were elevated in both groups of HFD‐fed rats (Sham and tSCI) in comparison to LFD‐fed animals, *P* (diet) < 0.05 (Table 1).

In order to determine if fatty infiltration of the skeletal muscle was occurring, we measured triglycerides and cholesterol in the skeletal muscle. Whereas there were no differences in the triglyceride and cholesterol content between Sham‐HFD and tSCI‐HFD, there was a significant reduction of triglycerides and cholesterol in tSCI‐LFD in comparison to Sham‐LFD, *P* (diet) < 0.05 (Table 1).

## Discussion

Spinal cord injury results in debilitating impact on motor and sensory control of the limbs. The long‐term reductions in mobility and locomotor activity result in negative impact on the metabolic health of injured persons (Smith and Yarar‐Fisher 2016). Even beyond the altered energy expenditure complexities following SCI, changes in energy intake and utilization may contribute to the increased MetS rates in this population (Smith and Yarar‐Fisher 2016). Chronic injury to the cord can also alter the functioning of the central nervous system as whole, changing the neural connectivity within and between higher‐order brain centers and germane to the current work, body‐weight regulation (Smith and Yarar‐Fisher 2016). Finally, in the context of nutrient rich, palatable Western diets that are both high in saturated fats and carbohydrates, persons with SCI may fare worse than noninjured individuals because of the various neural and hormonal impairments associated with their injury (Smith and Yarar‐Fisher 2016).

In the present work, we used a rodent model of thoracic‐10 level spinal contusion compared to Sham laminectomy‐operated rats to determine whether two types of macronutrient compositions, LFD versus HFD would alter metabolic outcomes in rats with the various end‐point measures culminating 16 weeks postinjury (12 weeks on the special diet). We probed energy intake, energy expenditure by metabolic monitoring. We predicted that under HFD conditions, SCI rats would have significantly increased metabolic dysfunction over LFD‐fed SCI rats.

### Body‐weight regulation following spinal cord injury: body weight gain

A robust body of work has documented that significant weight loss occurs with thoracic lesions in rats. Lesions that are high thoracic (T3) and complete will typically recover presurgical body weight within 10 days of injury (Primeaux et al. 2007; Ramsey et al. 2010) but body weight may permanently be reduced in comparison to uninjured rats (Primeaux et al. 2007). Using a T9‐10 contusion model, rats recover presurgical body weight around 14–21 days postinjury (Jeong et al. 2011; Gaudet et al. 2019). However, this may vary not only with the strain of the rat but also sex (Gaudet et al. 2019). The male Long Evans rats recovered presurgery body weight between 14 and 21 days postinjury and then continued on their growth trajectory never experiencing catch‐up growth; this is in line with the rodent literature (Jeong et al. 2011; Gaudet et al. 2019). Taken together, the first month of recovery of the rats is in line with data in the field.

Dietary manipulations to improve health and provide neuroprotection are of great interest in the SCI field. Use of specialized diets rich in nutrients such as the DHA, EPA, and choline such as Fortasyn^®^ diet have been administered to SCI rats to enhance motor recovery (Pallier et al. 2015). Given the recent public interest in ketogenic diets, the anti‐inflammatory and neuroregenerative potential of ketogenic diets have been applied to SCI research supplying rats with diets very high in fat and simultaneously low in carbohydrates (Streijger et al. 2013); these have shown beneficial results (Streijger et al. 2013). Still others have directly administered SCI rodents with omega‐3 or −6 fatty acids in hope of enhancing neuroprotection following injury (King et al. 2006; Lim et al. 2013). To our knowledge, our previous microarray study was the first study where tSCI animals were placed on a western‐style, high‐fat, high‐carbohydrate diet chronically (8 weeks) to exacerbate metabolic dysfunction, and obesity (Spann et al. 2017). In the current study, we again utilized this western‐style HFD but also in parallel, expanded the study to include a control LFD; we extended use of the diet to a total of 12 weeks to maximize its effects. Despite the significant body weight loss during the first several weeks following injury, when placed on HFD, the tSCI animals accelerated their weight gain but not as drastically when consuming LFD; this is in direct contrast to the Sham rats who equally put on weight with these diets. Body weight gain in the SCI rats consuming HFD tracked far more consistently with the Sham rats on HFD than did the body weight gain of tSCI rats on the LFD. From these data, tSCI animals may be more susceptible to weight gain when fed a diet of high fat/high carbohydrates than when fed a LFD; this is exactly what we hypothesized.

### Body‐weight regulation following spinal cord injury: food intake

In some studies, SCI individuals have been shown to consume a greater amount of calories when compared with individuals that have had other injuries rendering them less mobile (Pellicane et al. 2013). We did not observe differences in cumulative food intake in tSCI animals when directly comparing them to Sham animals maintained on the same diet. The only differences in the amount of calories consumed were group differences between LFD and HFD. This is expected since HFD is more calorically dense than LFD (4.54 kcal/g vs. 3.81 kcal/g, respectively).

Shifts in the type of macronutrients consumed by injured persons have also been reported; SCI individuals are reported to consume higher levels of fats and simple carbohydrates (as opposed to complex carbohydrates) (Sabour et al. 2012). Another report suggests that persons with SCI consume far more protein and carbohydrates then recommended by the USDA (Farkas et al. 2019). We specifically used the macronutrient selection test to determine if there was a preference by tSCI rats to consume a particular macronutrient. This test has been used successfully in other models (Wilson‐Perez et al. 2013). The stark difference in macronutrient preference that we observed in tSCI animals was a marked preference for protein in comparison to Sham rats. With the reduced lean (muscle) mass due to atrophy that is clearly hallmark of SCI, there may be an increased physiologic drive to increase protein ingestion to stave muscle loss. This could be further explored in future studies.

### Body composition changes following SCI

The thoracic spinal cord injury has a significant impact on lean body mass that persists to 16 weeks postinjury. This is in line with previous reports in rats which show reduced lean mass in T3‐lesioned rats by NMR but then no difference in lean body mass composition when normalized to body weight (Primeaux et al. 2007). In SCI individuals, the loss of lean body mass can lead to a 50% reduction in the skeletal cross‐sectional area in comparison to able‐bodied controls (Castro et al. 2000). This atrophy is only partially from peripheral denervation of the muscles but predominantly from reduced muscle loading/unloading and movement disuse (Bauman and Spungen 2000). This reduced muscle mass is consistent in the tSCI rat for the course of the study. Beyond the mass of the muscle, the quality of the muscle is also altered in SCI; infiltration of fat is high resulting in sarcopenic obesity. In this study, tSCI rats consuming LFD had reduced accumulations of triglycerides and cholesterol within the muscle in comparison to Sham‐LFD. The HFD‐fed tSCI rats had equivalent levels of triglycerides and cholesterol to the Sham‐HFD‐fed rats. So even in the rodent, reduction in lean mass coupled with consuming a HFD contributes to the sarcopenic obesity observed in this population.

Because standard laboratory chow cannot be matched in micronutrients or ingredients to the butterfat HFD, for this current study, we intentionally used the manufacturer‐suggested, nutrient‐matched “control” LFD consisting of 9% butterfat. This palatable LFD diet also has great obesogenic potential as can be well observed by the increase in body weight and adiposity of the Sham‐LFD rats. After 16 weeks, both diets causes substantial increases in body fat mass. Although this can be viewed as a weakness of the current study in that we did not have a lean control group, on the other hand, the strength of the study is that we heavily controlled for the micronutrient but not the macronutrient percentage under which the adiposity developed. Adiposty developed either through high fat or high carbohydrates. The variability of adiposity gain on an individual level in the current study reduced our ability to see group differences. Nonetheless, HFD consumed by tSCI rats clearly results in gains in fat mass composition in the injured animals that is akin to the adiposity gain of the Sham animals. In the human population, fat mass in the lower body measured by DEXA increases substantially following injury (Singh et al. 2014). In this study, we did not measure changes within the specific depots of fat. We conjecture that visceral and mesenteric adiposity was increased in the tSCI rats. Future work will necessitate DEXA analysis of the rats over time to determine depot specific changes.

Rats with a T9 or 10 spinal contusion have remarkable return of locomotor function as evidenced by improvements of BBB scores during the course of the 16‐week period of this study BBB scores reflect the locomotor function of the rat at the time of injury and return of mobility. However, the field of spinal cord injury uses a vast array of methods to produce injury from complete lesions to contusions using impactors, to ball‐drop contusions. In a T3 complete lesion, for instance, even after 18 weeks, BBB scores may not surpass a 4 (Primeaux et al. 2007). In the early weeks of a T8 contusive lesion, BBB scores may range between 5 and 8 (Vasconcelos et al. 2016). On the other hand, early scores of rats with a T9‐10 lesion begin at a 0 and can improve to 12–15 in a matter of weeks (Mills et al. 2001; Gaudet et al. 2017). This remarkable recovery within the rat model does often diminish its translatability to the human condition where recovery of function is very slow. However it does allow us to study the immediate effects of spinal cord injury and the effects of diet during recovery period.

### Body‐weight regulation following spinal cord injury: energy expenditure

We placed animals in the metabolic cages to assess various components of energy expenditure starting at week 9 on diet (13 weeks after injury). tSCI animals had significantly increased heat production resulting in increased energy expenditure calculations. The tSCI rats also had reduced locomotor activity in the metabolic cages. The reduced mobility exhibited by the tSCI animals may require that more energy be used for thermogenesis to maintain body temperature. We did not measure body temperature during these experiments but would predict body temperature would be similar to Shams. Body temperature for paraplegics is typically more akin to able‐bodied individuals whereas core temperature is more unstable and variable in tetraplegics (Thijssen et al. 2011).

### Circulating analytes of metabolic disease

In general, the circulating analytes that are typically elevated with MetS are increased in the HFD‐fed animals irrespective of whether they are Sham or tSCI. Leptin and insulin, peripheral markers of adiposity and glycemic control are increased in the animals fed a HFD. Nonfasted triglyceride levels are very high as are fasted cholesterol levels in the HFD‐fed animals. All these are indicative of long‐term consumption of an obesogenic diet, high in saturated fat.

### Summary

SCI partially altered energy balance through reduced mobility. Initially, as innervation to the limbs is compromised, lean and fat mass is lost reducing overall body weight as locomotor activity is reduced. Despite the potential of the LFD to produce obesity, the lower fat and higher carbohydrate content preserves a reduced feed efficiency and lipid content within the muscle of tSCI rats. However the high‐butterfat diet accelerates metabolic dysregulation for the SCI animals. This suggests that lipid metabolism may be affected in SCI rats particularly with higher fat loads. More work needs to be performed to determine how this occurs.

## Conflict of Interest

There are no conflict of interest, financial or otherwise, for K.K.H, A.R.H, B.C.D, R.J.G, or B.E.G.
